# Supplementary material for: CRISPR/Cas9-mediated knockout of the ubiquitin variant UbKEKS reveals a role in regulating nucleolar structures and composition
Source: Biol Open. 2023 Sep 21;12(9):bio059984. doi: 10.1242/bio.059984 (PMC10537958; doi:10.1242/bio.059984)
Supplement: Supplementary information [file biolopen-12-059984-s1.pdf]

A

Total Cell Extracts Spectral library

Cell lines	Mix of HeLa / U2OS / HEK293 / HCT116
Type of sample	Total cell extracts
DDA raw files	145
Protein FDR	5 %
Identified peptides	132 401
Identified proteins	9 122

B

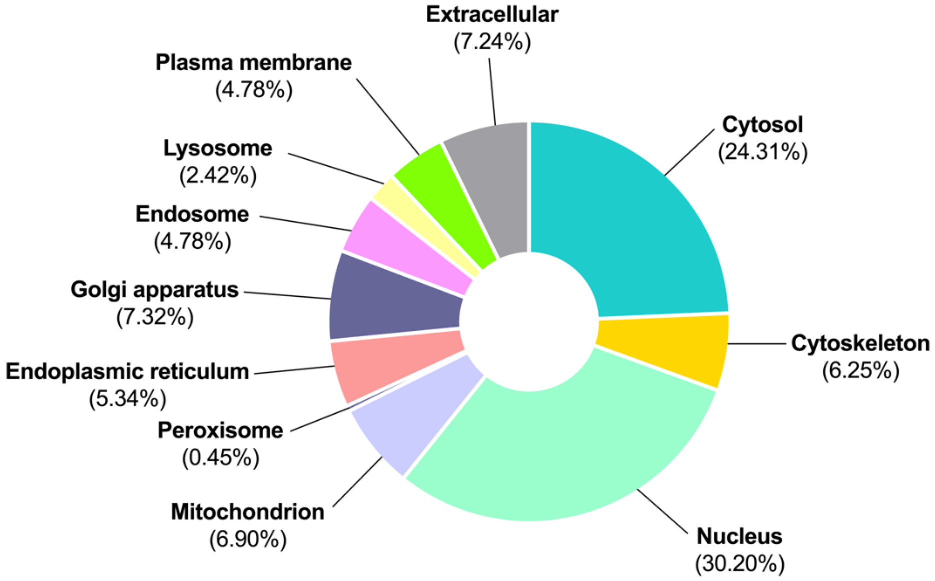

Fig. S1. Total cell extracts spectral library.

A. Main characteristics of the spectral library generated for total cell extracts DIA mass spectrometry. **B.** Representative distribution of proteins identified during spectral library generation. Location was determined using the COMPARTMENTS prediction tool with Cytoscape. Only locations with a false discovery rate lower than 5% were considered.

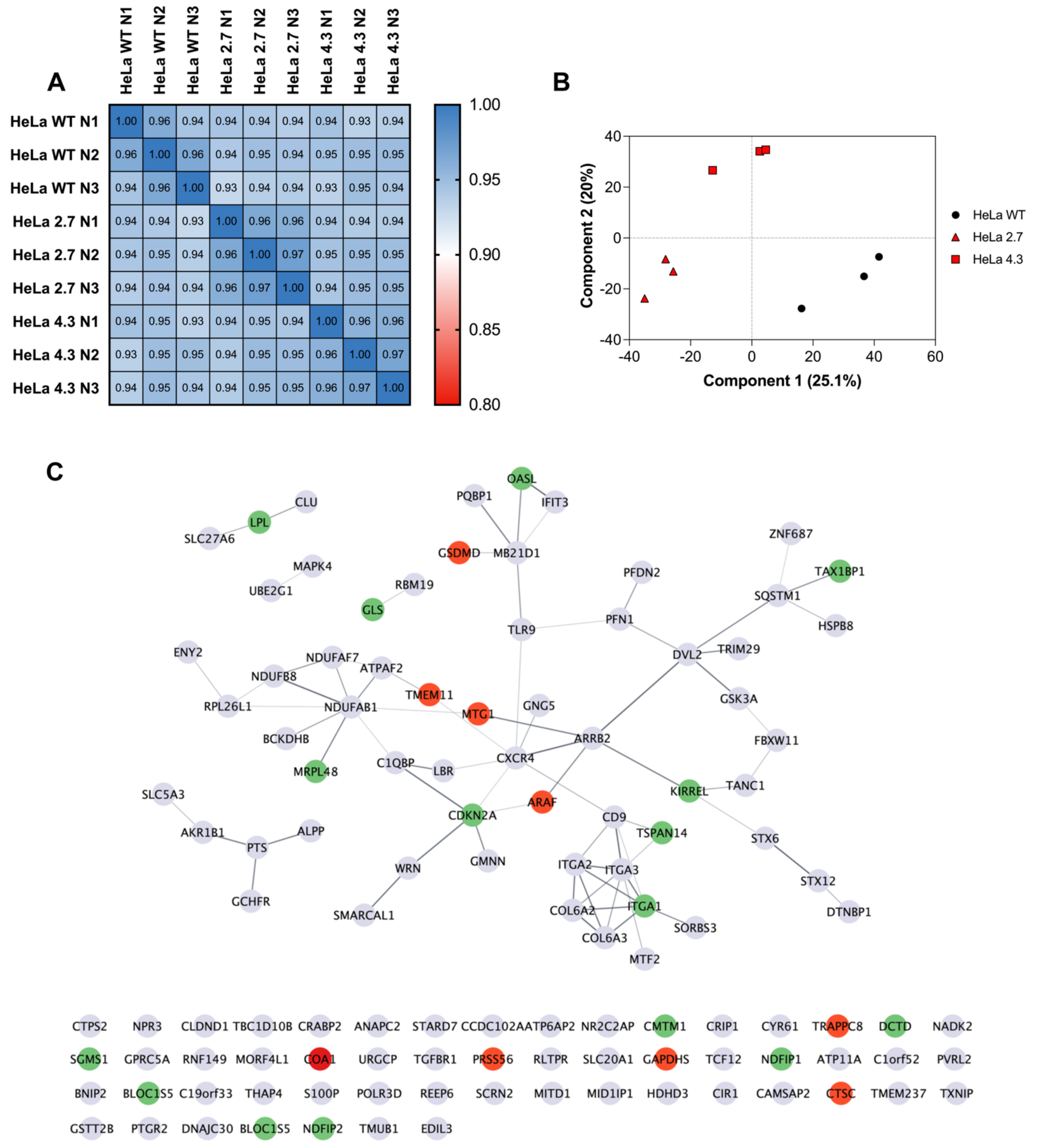

Fig. S2. Complementary data for the DIA run on total cell extracts. A. Pearson's correlation matrix for each replicate of the experiment (n=3 independent experiments, each read twice by the mass spectrometer). **B.** Principal Component Analysis (PCA) using Benjamini-Hochberg cut-off algorithm (false discovery rate = 5%, n=3 independent experiments, each read twice by the mass spectrometer). **C.** Interactions network of proteins significantly downregulated or upregulated in Ub^{KEKS} knockout cells. A complete list of these proteins is available in Table S1. Downregulated and upregulated proteins which are common to both HeLa 2.7 and HeLa 4.3 are coloured in red and green respectively. A functional enrichment analysis was also performed with the online tool ShinyGO (version 0.76) using False Discovery Rate cut-offs of 0.05 combined with the GO biological Process Pathway database (n=3 independent experiments, each read twice by the mass spectrometer). No significant functional enrichment was retrieved using these parameters (not shown).

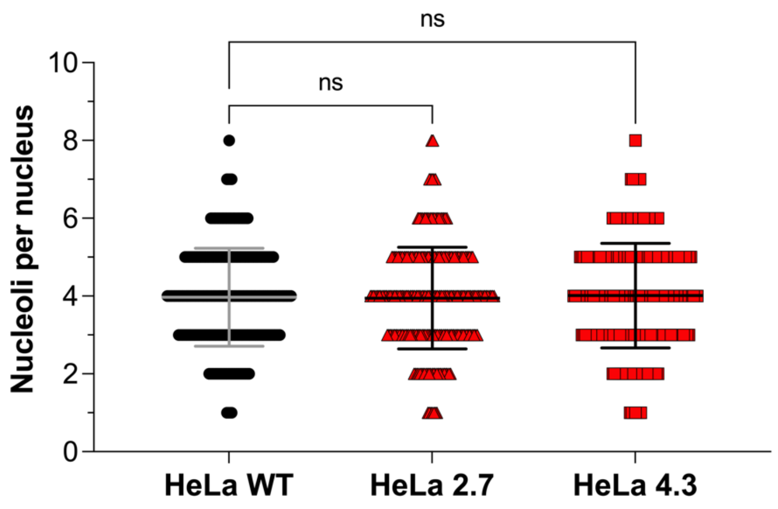

Fig. S3. Loss of Ub^{KEKS} does not impact the number of nucleoli in the cell.
The number of nucleoli for each nucleus was counted with Fiji (version 1.53c) software, using nucleolin staining presented in Fig. 3. Centre line indicates the means of each population while error bars show standard deviations. Statistical analysis was performed using one-way ANOVA and Dunnett's multiple comparisons post hoc test. Adjusted p-values for comparison of wild type HeLa cells with Ub^{KEKS} knockout cells are respectively equal to 0.9694 for HeLa 2.7 clones and to 0.9044 for HeLa 4.3 clones (n> 250 nuclei examined over four independent experiments).

Biology Open • Supplementary information

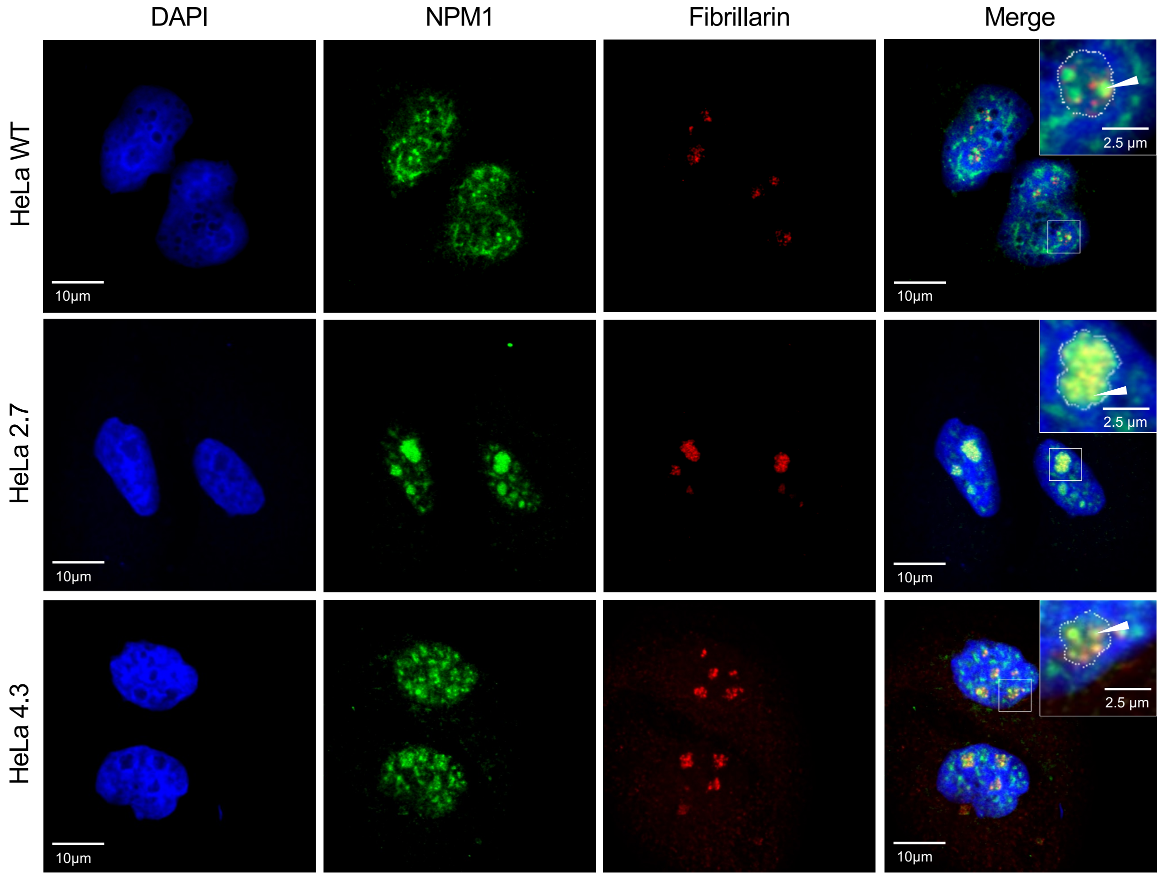

Fig. S4. Granular Component and Dense Fibrillar Component dual-labelling in wild type and Ub^{KEKS} knockout HeLa cells.

Granular Component and Dense Fibrillar Component were labelled using endogenous NPM1 and Fibrillarin antibodies respectively. DAPI was also used to stain nuclei. Enlarged areas are indicated by a white box. Granular Component's edges (i.e., the edges of the nucleolus) are outlined by a dotted line. Concentric substructure Dense Fibrillar Components are indicated by a white arrow. Scale bars in main images correspond to 10 µm. Scale bars for enlarged regions of interest (upper right corner of the last panel) indicate 2.5 µm. A graphical representation of nucleolar structures is available in Fig. 2A (n=1, for illustrative purpose).

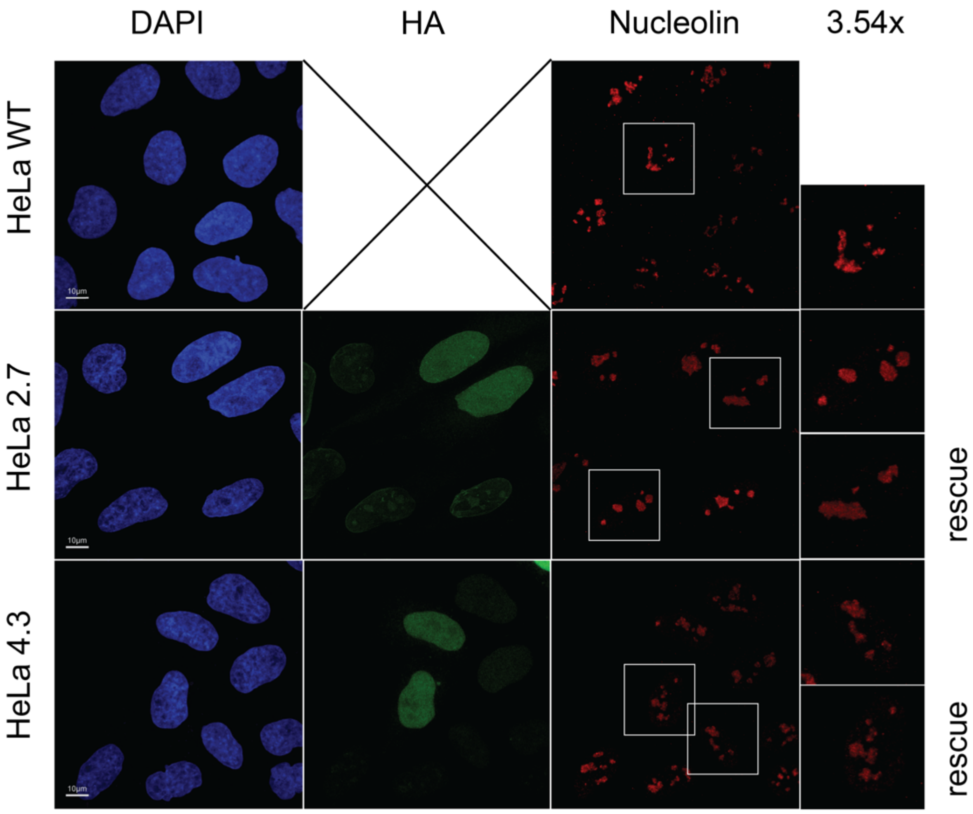

Fig. S5. Ub^{KEKS} overexpression in knockout cells rescue the nucleoli's circularity phenotype. Wild type and Ub^{KEKS}-HA transfected knockout HeLa cells were labelled for immunofluorescence microscopy using HA and Nucleolin antibodies. Nuclei were also stained with DAPI. Enlarged regions (right panel) of untransfected and rescued cells (i.e., HA-positive cells) are indicated by a white box. Scale bars correspond to 10 µm. Complete nucleolus circularity measurements and statistical analysis are available in Fig. 3 (n=4 independent experiments).

A

Nucleoli Spectral library	
Cell line	HeLa
Type of sample	Isolated nucleoli
DDA raw files	10
Protein FDR	1 %
Identified peptides	29 928
Identified proteins	3 418

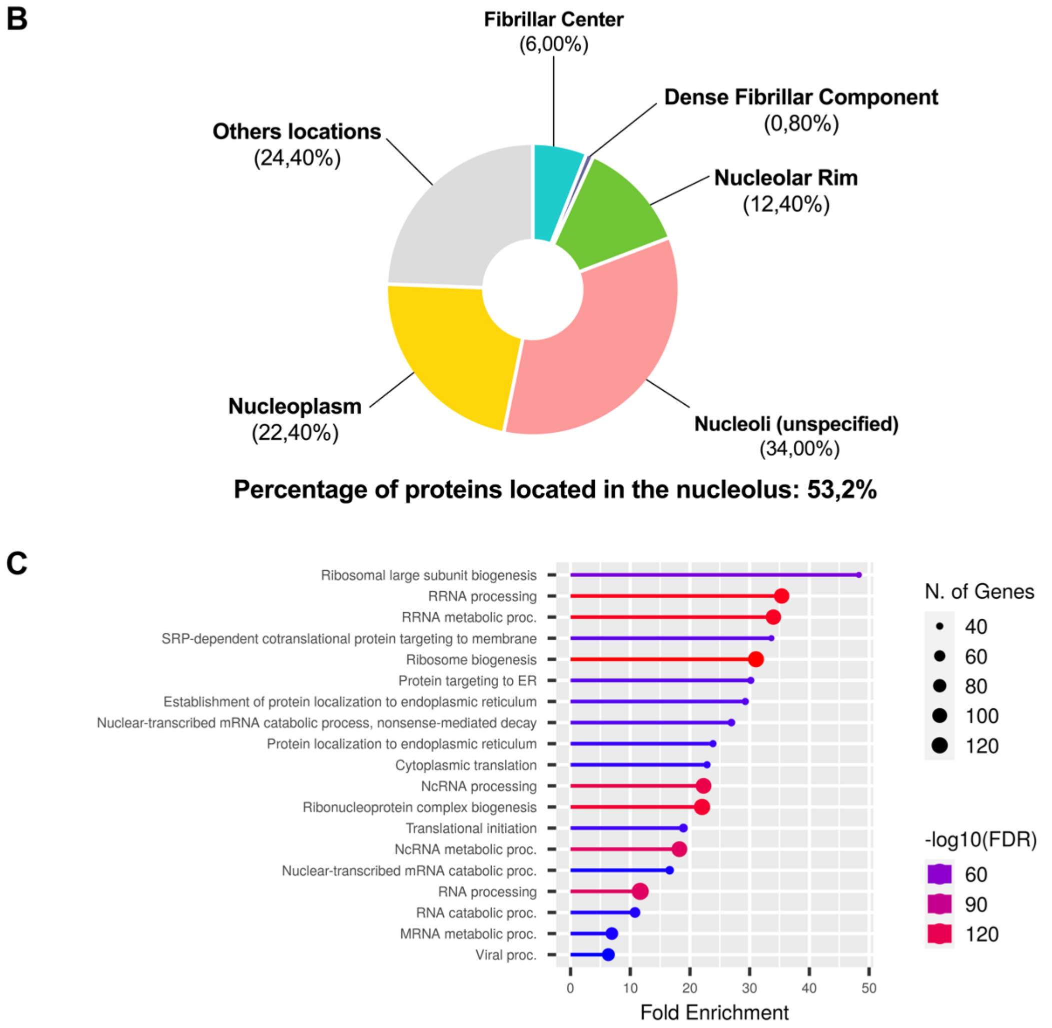

Fig. S6. Nucleolar spectral library.
A. Main characteristics of the spectral library generated by DIA mass spectrometry from isolated nucleoli. **B.** Representative distribution of the 250 most abundant proteins identified during spectral library generation. A complete list of these proteins is available in Table S2. Location was determined using the COMPARTMENTS prediction tool with Cytoscape. Only locations with a false discovery rate below 5% were considered. **C.** Functional enrichment for the 250 most abundant proteins of the nucleolar spectral library. Analysis was performed via the online tool ShinyGO (version 0.76) with False Discovery Rate cut-offs of 0.05 using the GO biological Process Pathway database.

Biology Open • Supplementary information

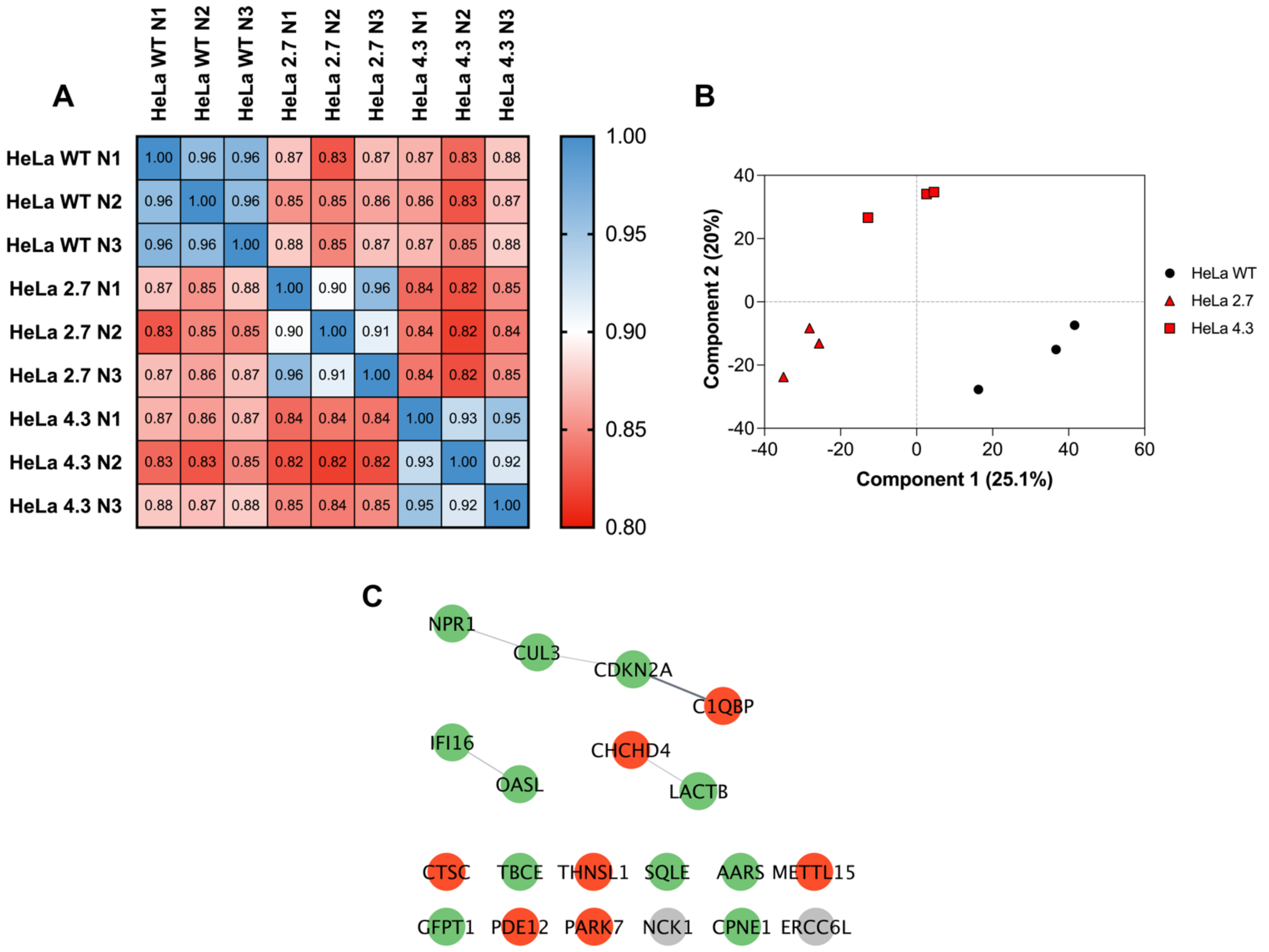

Fig. S7. Complementary data for the DIA run on isolated nucleoli. A. Pearson's correlation matrix for each replicate of the experiment (n=3 independent experiments, each read twice by the mass spectrometer). **B.** Principal Component Analysis (PCA) using Benjamini-Hochberg cut-off algorithm (false discovery rate = 5%, n=3 independent experiments, each read twice by the mass spectrometer). **C.** Interactions network of proteins significantly downregulated or upregulated in both Ub^{KEKS} knockout cells. A complete list of these proteins is available in Table S3. Downregulated and upregulated proteins which are common to both HeLa 2.7 and HeLa 4.3 are coloured in red and green respectively. Proteins in grey were significantly upregulated in HeLa 2.7 but significantly downregulated in HeLa 4.3 (n=3 independent experiments, each read twice by the mass spectrometer).

Table S1. Complete list of proteins significantly modulated in UbKEKS knockout cells.

In total, 74 proteins are downregulated and 48 proteins are upregulated in HeLa 2.7 and HeLa 4.3 total cell extracts. Table S1 provides the Uniprot identifier for each protein, along with a quick description and the associated parental gene. (n=3 independent experiments, each read twice by the mass spectrometer).

Click here to download Table S1

Table S2. List of the 250 most abundant proteins identified by DIA mass spectrometry in purified HeLa WT nuclei samples.

These proteins were identified during the nucleoli spectral library creation (Fig. S5) and used to assess the quality of the library (Fig. S5b and Fig. S5c). Table S2 provides the Uniprot identifier for each protein, along with a quick description, protein length and weight, and finally the associated parental gene. Mass spectrometry data such as detected intensity, MS/MS count, number of detected peptides and percentage of sequence coverage are also available.

Click here to download Table S2

Table S3. List of proteins significantly modulated in the nucleoli of UbKEKS knockout cells.

In total, 53 proteins are downregulated and 57 proteins are upregulated in HeLa 2.7 and HeLa 4.3. Table S3 provides the Uniprot identifier for each protein, along with a quick description and the associated parental gene. (n=3 independent experiments, each read twice by the mass spectrometer).

Click here to download Table S3

Biology Open • Supplementary information
